# Differences in Mortality by Donor Sex and Age in a Multinational Cohort of Liver Transplant Recipients

**DOI:** 10.1097/TXD.0000000000001912

**Published:** 2026-02-11

**Authors:** Lauren T. Grinspan, Xun Zhang, Mourad Dahhou, Caner Süsal, Bernd Döhler, Anette Melk, Amanda Vinson, Bethany J. Foster

**Affiliations:** 1 Recanati/Miller Transplantation Institute, Icahn School of Medicine at Mount Sinai, New York, NY.; 2 Research Institute of the McGill University Health Centre, Montreal, QC, Canada.; 3 Institute of Immunology, Heidelberg University Hospital, Heidelberg, Germany.; 4 Transplant Immunology Research Center of Excellence TIREX, Koç University, Istanbul, Turkey.; 5 Koç University School of Medicine, Istanbul, Turkey.; 6 Children’s Hospital, Hannover Medical School, Hannover, Germany.; 7 Nephrology Division, Department of Medicine, Dalhousie University, Halifax, NS, Canada.; 8 Department of Epidemiology, Biostatistics, and Occupational Health, McGill University, Montreal, QC, Canada.; 9 Division of Nephrology, Department of Pediatrics, McGill University Faculty of Medicine, QC, Canada.

## Abstract

**Background.:**

Prior studies assessing the association between donor sex and liver transplant outcomes showed conflicting results. We hypothesized that donor age, recipient sex, and time posttransplant modify the relationship between donor sex and mortality.

**Methods.:**

First deceased donor liver transplant recipients 13 y or older recorded in the Scientific Registry of Transplant Recipients and the Collaborative Transplant Study (n = 198 856; 1988–2019) were studied. We used multivariable Cox models to estimate the association between donor sex and mortality, accounting for the modifying effects of recipient sex, donor age (13–44, 45–59, ≥60 y), and time posttransplant (<3 versus ≥3 mo). Results from cohort-specific Cox models were combined using a 2-stage individual-patient data meta-analysis.

**Results.:**

Among male recipients, early posttransplant mortality was higher with female than male donors (only statistically significant with donors aged 13–44 y); subsequently, mortality was higher with female than male donors aged 13–44 y but *lower* with female than male donors aged 45 y or older. Among female recipients, early posttransplant mortality was lower with female than male donors (only statistically significant with donors aged 45–59 y); subsequently, there were no significant differences in mortality by donor sex. Donor sex-related differences in mortality appeared to be driven by differences in graft survival. In the first 3 mo posttransplant, absolute mortality rates were higher in the Collaborative Transplant Study compared with the Scientific Registry of Transplant Recipients.

**Conclusions.:**

Donor age modifies the association between donor sex and mortality. Among male recipients, young female donors were associated with higher mortality than young male donors, but female donors older than 45 y may offer superior long-term patient survival than same-aged male donors.

## INTRODUCTION

Understanding the independent association between donor sex and liver transplantation outcomes may be helpful in developing personalized posttransplant management plans, optimizing organ preservation strategies, and understanding the mechanisms by which donor sex may affect graft outcomes in other organ transplants. In kidney transplantation, the association between donor sex and graft outcomes is modified by donor age such that older female donors may offer slightly better outcomes than older male donors.^1^ In liver transplantation, the data are mixed. Some studies suggested a difference in liver graft failure risk by donor sex. Since the first study in 1993 that found poorer outcomes for male recipients of female donors compared with other donor-recipient sex combinations,^2^ several others have reported similar findings.^3-7^ However, these studies neither adjusted for donor age nor considered donor age as an effect modifier. A 2011 study of deceased and living donor liver recipients recorded in the Scientific Registry of Transplant Recipients (SRTR) found no differences in graft outcomes by donor sex among male recipients after adjustment for potential confounders^8^; no comparisons by donor sex were made for female recipients. A 2002 study of deceased donor liver recipients in the Collaborative Transplant Study (CTS) also found no differences in the risk of death or graft loss by donor sex among male or female recipients.^9^ However, neither of these studies considered effect modification by donor age.

More recently, a single-center study limited to 309 male liver transplant recipients showed a significant interaction between donor sex and donor age: the risk of death or retransplant was significantly higher with a female than male donor when the donor was aged 40 y or younger but did not differ by donor sex when the donor was older than 40 y.^10^ This study suggested that donor age may modify the effect of donor sex on liver transplant outcomes but was limited by a small sample and the absence of female recipients. We aimed to compare mortality rates between recipients of male and female donors, accounting for the modifying effects of recipient sex and donor age, in 2 of the largest transplant databases worldwide: the US SRTR and the international CTS database. This analysis also allows a comparison of mortality rates across these 2 large databases.

## MATERIALS AND METHODS

### Data Sources and Population

This was a retrospective cohort study of recipients aged 13 y or older of a liver-only transplant from a deceased donor aged 13 y or older recorded in the SRTR or the CTS database. The SRTR cohort included patients transplanted between January 1, 1988, and December 31, 2018, and followed until June 1, 2019. Observation ended December 31, 2019, to avoid the impact of the COVID-19 pandemic, which led to a major increase in mortality among transplant recipients and affected male and female individuals differently.^11^ Observation was censored at retransplant, end of observation, or end of the study period. Observation was limited to the first transplant because donor characteristics will change with subsequent transplants. The study was approved by the McGill University Health Center Research Ethics Board (No. 2020-6264). Patients recorded in the SRTR are not required to provide informed consent; this is a mandatory, national registry. The transplant centers participating in CTS certify that all patients provided written informed consent for the transfer of their data to the CTS.

The SRTR data system includes data on all donors, waitlisted candidates, and transplant recipients in the United States, submitted by the members of the Organ Procurement and Transplantation Network. The Health Resources and Services Administration, US Department of Health and Human Services, provides oversight to the activities of the Organ Procurement and Transplantation Network and SRTR contractors. The CTS is 1 of the largest voluntary international medical registries.^1,12^ The CTS includes data from >400 centers in 42 countries. We excluded countries with <100 transplants or <10 patients under observation at 10 y; only 2% of transplants reported in the CTS were excluded on the basis of these criteria. Included countries are listed in **Supplemental Data** (**SDC,** https://links.lww.com/TXD/A830).

### Outcome and Exposure Definitions

The primary outcome was patient death. The primary exposure was donor sex. The relationship between donor sex and mortality risk is likely to differ by recipient sex^13^; therefore, we considered donor-recipient sex combinations: female donor-female recipient, male donor-female recipient, female donor-male recipient, and male donor-male recipient. This approach allowed us to investigate the association between donor sex and mortality from the perspective of a female recipient separately from the perspective of a male recipient. We also included a donor-recipient sex by donor age interaction since the association between donor sex and graft outcome differed by donor age in kidney transplantation^1^ and prior work suggested this was also likely in liver transplant.^14^ We considered the possibility that the association between donor sex and mortality may differ by recipient age, but there was no significant interaction between donor-recipient sex combination and recipient age. Therefore, subsequent models were adjusted for recipient age but did not include a donor-recipient sex combination by recipient age interaction. Observation was censored at retransplant.

### Data Harmonization

The distributions of all variables to be included in the models were examined within each cohort. Where differences existed between the 2 databases, new, harmonized variables were generated to ensure uniform representation of each variable across cohorts. For example, harmonized primary disease categories were created.

### Statistical Analysis

Analyses were first performed for each cohort separately. Results were then combined across the cohorts, weighted for observation time, using 2-stage individual patient data random-effects meta-analyses.^1,15,16^

### Association Between Donor Sex and Survival

Cox proportional hazards models were used to estimate the association between donor sex and mortality. Because hazards were not proportional over time, we included a time-varying time posttransplant variable (first 3 mo versus time subsequent to 3 mo). This allowed separate hazard ratios (HRs) to be estimated for the immediate posttransplant period (0–3 mo) and long-term follow-up (>3 mo). We also included a donor-recipient sex by donor age (13–44 y, 45–59 y, 60 y and older; categories based on developmental stages of reproductive age, postreproductive age, older age, respectively) interaction. Unadjusted analyses were followed by multivariable analyses adjusted for factors that could confound the relationship between donor sex and mortality based on their known association with graft or patient outcomes.^8,17-20^ Potential confounders included: recipient race, primary disease, cold ischemia, transplant year, reduced liver versus whole, donor-recipient weight ratio (SRTR only; used as a measure of size match between donor and recipient), donor hypertension, donor cytomegalovirus, donor cerebrovascular accident as cause of death, and age at transplant. Age at transplant was treated as a piecewise linear variable; this allows for different slopes in the relationship between the variable and the outcome. In this case, we included 3 different slopes in the relationship between recipient age and mortality: 13–<20 y, 20–<35 y, and ≥35 y. These pieces were based on the shape of the association between recipient age at transplant and mortality. Missing values were imputed (15 imputations) using the fully conditional specification method; parameter estimates were pooled across the imputed data sets as per Rubin’s rule.^21^ This imputation method uses the joint distributions of all variables included in the model to impute missing values and incorporates the uncertainty introduced by missing data into the calculation of confidence intervals (CIs).

Adjusted HR (aHR) is expressed as the hazard for recipients of a female relative to a male donor and presented with 95% CIs. Proportionality was assessed by refitting the models, censoring all observations at 5 and 10 y. HR were unchanged, indicating that hazards were proportional.

The cohort-specific HR were pooled on the basis of a weighted average of the effect sizes of the cohorts using random-effects models.^1,22,23^ Random-effects models allow for the possibility that observed aHR estimates may vary across cohorts due to real differences in the association between donor sex and mortality within each cohort or due to sampling variability. Heterogeneity in outcome between cohorts was evaluated by Higgins *I*^2^ and the chi-square test of heterogeneity.

### Sensitivity Analysis

Because the donor:recipient weight ratio was not available in the CTS, SRTR models were refitted excluding this variable to determine the impact its exclusion of this variable on the point estimates associated with donor sex.

### Comparison of Graft Failure by Donor Sex

To better understand how differences in graft failure rates by donor sex contribute to the observed differences in mortality by donor sex, we also compared graft failure (defined as retransplant or death due to graft failure) between female and male recipients of female and male donors in each donor age category. This analysis was conducted only in the SRTR cohort; it was not possible to accurately capture deaths due to graft failure in the CTS cohort. To allow direct comparisons with the primary analysis, this analysis also included an interaction by time posttransplant (first 3 mo and time subsequent to 3 mo posttransplant).

Data analyses were performed using Statistical Analysis System version 9.4 (SAS Institute, Carry, NC) and R version 4.2.0.

## RESULTS

### Patient Characteristics

We identified 127 118 recipients in the SRTR (34.6% female individuals) and 71 738 recipients in the CTS (34.0% female individuals) who met inclusion criteria. There were 48 465 deaths (38.1%) >5.0 (interquartile range [IQR], 1.6–10.3] y of follow-up in the SRTR and 22 940 deaths (32.0%) >4.0 (IQR, 1.0–9.0) y of follow-up in the CTS. An overview of the characteristics of each cohort is presented in Table 1. A higher proportion of donors was male than female in both SRTR (60.2%) and CTS (56.3%). Primary disease distributions were also similar for SRTR and CTS. Donors in SRTR (median 40 y [IQR, 25–53]) were younger than in CTS (49 y [IQR, 34–61]). Cold ischemia time was lower in the SRTR than in the CTS, although there were more missing values in the CTS. There were more partial livers used in CTS than in SRTR (4.5% versus 1.4%). Hypertension was more common among donors in the SRTR than CTS (33.3% versus 11.1%). There were more donors after circulatory death in SRTR than CTS (3.2% versus 1.3%), although the vast majority of donors in both groups were not donors after circulatory death.

**TABLE 1.** Overview of patient characteristics (n, %)

	SRTR	CTS
Patients, N	127 118	71 738
Censoring (%)		
Death	48 465 (38.1)	22 940 (32.0)
Retransplant	8355 (6.6)	6627 (9.2)
Follow-up, y, median (IQR)	5.0 (1.6–10.3)	4.0 (1.0–9.0)
Age at transplant, y, median, IQR	54 (47–61)	53 (45–60)
Recipient race (%)		
White	109 915 (86.5)	36 186 (93.8)
Black	10 599 (8.3)	528 (1.4)
Other	6644 (5.2)	1853 (4.8)
Missing		33 176 (46.2)
Donor-recipient sex (%)		
Female donor → female recipient	21 081 (16.6)	13 621(19.0)
Female donor → male recipient	29 492 (23.2)	17 758 (24.8)
Male donor → female recipient	22 909 (18.0)	10 760 (15.0)
Male donor → male recipient	53 636 (42.2)	29 599 (41.3)
Primary disease (%)		
Congenital/biliary atresia	1078 (0.9)	1309 (1.8)
Alcohol associated liver disease	21 054 (16.6)	12 031 (16.8)
Liver tumors	17 725 (13.9)	10 256 (14.3)
Metabolic liver disease	3427 (2.7)	2231 (3.1)
Fulminant liver failure	6808 (5.4)	5408 (7.5)
Autoimmune conditions	16 255 (12.8)	9956 (13.9)
Hepatitis C	35 738 (28.1)	19 787 (27.6)
Others	25 033 (19.7)	10 760 (15.0)
Donor age, y, median, IQR	40 (25–53)	49 (34–61)
Transplant year (%)		
1988–1994	13 222 (10.4)	7259 (10.1)
1995–1999	15 729 (12.4)	10 550 (14.7)
2000–2004	19 680 (15.5)	13 262 (18.5)
2005–2009	23 890 (18.8)	14 442 (20.1)
2010–2014	24 284 (19.1)	14 425 (20.1)
2015–2019	30 313 (23.9)	11 820 (16.5)
Cold ischemia time, h (%)		
<8	76 820 (64.0)	23 778 (38.7)
8–<12	32 288 (26.9)	27 578 (44.9)
≥12	10 851 (9.1)	10 007 (16.3)
Missing	7159 (5.6)	11 691 (14.9)
Donor-recipient weight ratio (%)		
≥0.9	69 724 (57.3)	
<0.9	51 890 (42.8)	
Missing	5504 (04.3)	
Graft size (%)		
Whole	125 358 (98.6)	68 541 (95.5)
Reduced	1760 (1.4)	3197 (4.5)
Donor CMV (%)		
Positive	80 062 (63.4)	28 356 (54.8)
Negative	46 235 (36.6)	23 343 (45.2)
Missing	821 (0.6)	20 039 (27.9)
Donor cause of death (%)		
Cerebrovascular accident	78 295 (61.6)	39 363 (59.9)
Other	48 737 (38.4)	26 365 (40.1)
Missing	86 (0.1)	6010 (8.4)
Donor hypertension (%)		
Yes	38 247 (33.3)	7360 (11.1)
No	76 733 (66.7)	58 840 (88.9)
Missing	12 138 (9.5)	5538 (7.7)
Donor donation after circulatory death (%)		
Yes	4113 (3.2)	849 (1.3)
No	123 005 (96.8)	66 372 (98.7)
Missing	0 (0)	4517 (6.3)

Unit of analysis here is the patient.

CMV, cytomegalovirus; CTS, Collaborative Transplant Study; IQR, interquartile range; SRTR, Scientific Registry of Transplant Recipients.

Table 2 (observation time in the first 3 mo posttransplant) and Table 3 (observation time subsequent to 3 mo) summarize the composition of the observed experience for recipients of female and male donors, by recipient sex and donor age, pooled across SRTR and CTS. Regardless of recipient sex, a greater proportion of the observation time contributed by recipients of female donors than by recipients of male donors was from recipients of donors with positive cytomegalovirus status from recipients with donor:recipient weight ratio <0.9 (in SRTR only). In **Tables S1 and S2** (**SDC,** https://links.lww.com/TXD/A830), SRTR, and in **Tables S3 and S4** (**SDC,** https://links.lww.com/TXD/A830), CTS show the same information for each cohort separately for each observation time period.

**TABLE 2.** Composition of the contrasted experience, pooled across SRTR and CTS, by donor sex and donor age for female and male recipients within the first 3 mo posttransplant (proportion per 100 patient years)

Donor age	Female recipients						Male recipients					
	13–44 y		45–59 y		≥60 y		13–44 y		45–59 y		≥60 y	
Donor sex	Female	Male	Female	Male	Female	Male	Female	Male	Female	Male	Female	Male
Person-years of observation	3553	4948	2585	1719	1775	1028	4531	10 827	3749	5222	2580	3333
Death	1058	1497	805	603	556	330	1144	2532	1020	1316	766	964
Age at transplant, y (%)												
13–<20	4.2	4.5	2.1	1.9	0.8	0.7	2.6	1.8	1.3	0.6	0.3	0.2
20–<35	11.1	10.9	8.7	8.4	5.2	5.1	6.5	6.0	5.1	4.2	2.9	2.3
≥35	4.2	4.5	2.1	1.9	0.8	0.7	2.6	1.8	1.3	0.6	0.3	0.2
Recipient race (%)												
White	85.5	85.9	87.0	86.5	88.5	89.0	87.5	89.2	88.4	90.3	88.2	91.1
Black	8.7	9.1	8.0	8.6	6.2	6.7	6.5	6.0	5.9	5.5	5.6	4.7
Others	5.9	5.0	5.0	4.9	5.3	4.3	6.0	4.9	5.7	4.3	6.2	4.2
Missing	11.4	9.2	15.8	13.1	26.5	21.9	11.1	10.8	16.6	17.7	33.9	33.5
Primary disease (%)												
Congenital/biliary atresia	2.2	2.2	1.8	2.2	1.7	2.2	1.1	0.9	0.7	0.5	0.4	0.4
Alcohol	9.9	10.4	11.2	11.7	12.8	12.4	18.4	19.4	19.2	19.7	20.8	22.0
Liver cancer	7.9	7.4	9.1	8.8	12.4	12.4	15.1	14.6	17.0	18.1	23.1	22.5
Metabolic	2.9	3.5	2.6	2.6	1.9	2.9	3.1	3.1	3.0	2.6	2.2	2.5
Fulminant	9.9	10.4	9.0	10.8	6.9	7.4	4.4	3.8	4.0	3.0	2.8	2.3
Autoimmune	26.4	24.1	23.9	20.0	21.4	17.8	9.4	8.8	7.9	7.2	6.6	5.6
Hepatitis C	20.9	20.9	22.1	22.9	21.0	21.4	32.5	32.7	32.3	32.8	28.3	28.8
Others	19.9	21.2	20.3	21.0	21.8	23.5	16.1	16.7	15.8	16.1	15.7	15.9
Cold ischemia time, h (%)												
<8	57.0	57.9	59.0	62.5	62.8	62.6	60.0	58.3	58.7	59.6	60.7	59.8
8–<12	28.8	28.4	29.6	28.1	29.3	29.4	28.4	29.0	30.6	30.1	30.5	31.3
≥12	14.1	13.7	11.4	9.4	7.9	8.0	11.6	12.7	10.8	10.3	8.7	8.9
Missing	12.7	11.7	13.3	11.7	15.2	13.5	11.1	11.4	12.0	12.7	16.4	16.0
Transplant year (%)												
1988–1994	16.7	18.2	8.6	7.5	2.6	2.6	10.3	13.3	5.7	5.4	1.7	1.6
1995–1999	16.0	16.7	13.8	13.0	9.3	8.8	14.5	14.9	11.6	10.6	7.1	7.1
2000–2004	16.9	15.7	17.7	15.6	14.8	15.7	16.9	17.0	17.3	16.9	15.5	15.3
2005–2009	16.0	15.7	19.9	19.3	22.1	21.1	18.2	18.0	22.2	21.7	23.6	23.4
2010–2014	15.8	15.3	19.7	21.5	25.4	25.2	18.9	17.1	22.6	21.7	26.5	25.8
2015–2019	18.6	18.3	20.4	23.0	25.7	26.5	21.2	19.6	20.6	23.7	25.7	26.7
Donor:recipient weight ratio,^*a*^ (%)												
≥0.9	56.9	68.4	61.9	72.8	59.5	73.4	42.3	56.3	46.7	64.7	44.2	61.8
<0.9	43.1	31.6	38.1	27.2	40.5	26.6	57.7	43.7	53.3	35.3	55.8	38.2
Missing	5.8	5.9	4.2	2.7	3.0	2.0	4.0	4.6	2.7	2.8	2.1	2.4
Graft size (%)												
Whole	95.0	93.7	98.5	98.1	99.8	99.8	97.4	97.0	99.1	99.3	99.8	99.8
Reduced	5.0	6.3	1.5	1.9	0.2	0.2	2.6	3.0	0.9	0.7	0.2	0.2
Donor CMV (%)												
Positive	60.9	53.4	68.0	60.0	76.0	68.3	63.1	53.8	68.8	58.5	74.1	66.5
Negative	39.1	46.6	32.0	40.0	24.0	31.7	36.9	46.2	31.2	41.5	25.9	33.5
Missing	6.1	5.8	7.9	7.4	16.9	14.8	6.9	6.9	9.8	10.7	25.3	25.1
Donor hypertension (%)												
No	87.6	92.9	65.5	62.1	56.7	52.4	84.5	90.4	60.9	61.7	58.5	57.9
Yes	12.4	7.1	34.5	37.9	43.3	47.6	15.5	9.6	39.1	38.3	41.5	42.1
Missing	13.1	14.1	7.7	6.4	3.8	3.8	8.8	11.2	5.7	5.5	3.1	3.1
Donor cause of death (%)												
Cerebrovascular accident	63.6	73.2	50.5	52.3	51.2	50.5	64.7	71.5	49.9	53.6	55.2	54.6
Other	36.4	26.8	49.5	47.7	48.8	49.5	35.3	28.5	50.1	46.4	44.8	45.4
Missing	3.8	3.2	2.9	2.7	2.3	2.4	3.0	3.1	2.9	2.6	2.4	2.3
Donation after circulatory death (%)												
No	97.5	97.3	98.0	97.2	99.5	99.2	96.9	96.2	97.8	96.9	99.4	99.0
Yes	2.5	2.7	2.0	2.8	0.5	0.8	3.1	3.8	2.2	3.1	0.6	1.0
Missing	3.1	2.8	2.1	2.0	1.1	1.2	2.4	2.6	2.0	1.6	1.1	1.0

Because the unit of analysis was person-time, rather than person, the characteristics presented are weighted by a factor derived from the number of person-years of observation and number of events. For example, 2.2% of the person-years contributed by female recipients of female donors between 13 and 44 y were from people with congenital liver disease/ biliary atresia as the primary disease.

^*a*^SRTR only.

CMV, cytomegalovirus; CTS, Collaborative Transplant Study; SRTR, Scientific Registry of Transplant Recipients.

**TABLE 3.** Composition of the contrasted experience, pooled across SRTR and CTS, by donor sex and donor age for female and male recipients after first 3 mo posttransplant (proportion per 100 patient years)

Donor age	Female recipients						Male recipients					
	13–44 y		45–59 y		≥60 y		13–44 y		45–59 y		≥60 y	
Donor sex	Female	Male	Female	Male	Female	Male	Female	Male	Female	Male	Female	Male
Person-years of observation	113 177	157 944	71 715	44 096	39 401	22 074	124 549	317 220	92 975	125 519	54 211	67 850
Death	4264	6113	3114	2042	2123	1207	5700	13 985	4730	6641	3250	4186
Age at transplant, y (%)												
13–<20	4.2	4.6	2.1	2.2	0.9	0.6	2.9	1.9	1.5	0.7	0.5	0.2
20–<35	11.9	11.5	9.2	8.7	5.8	5.8	7.1	6.7	5.5	4.6	3.4	2.4
≥35	83.9	84.0	88.7	89.1	93.3	93.6	90.0	91.4	93.0	94.7	96.2	97.5
Recipient race (%)												
White	86.9	87.5	87.9	87.7	88.9	89.0	88.1	90.3	88.5	90.9	87.5	91.4
Black	7.8	8.2	7.3	7.6	5.8	6.6	5.8	5.4	5.2	4.8	5.3	4.0
Others	5.3	4.3	4.8	4.7	5.3	4.4	6.1	4.3	6.3	4.2	7.2	4.5
Missing	10.8	8.7	12.4	12.0	21.7	19.3	10.5	9.9	14.6	15.7	30.3	29.8
Primary disease (%)												
Congenital/biliary atresia	2.3	2.2	1.7	2.1	1.5	2.1	1.2	0.9	0.7	0.5	0.5	0.4
Alcohol	7.9	8.4	9.6	9.7	11.8	10.9	17.2	18.2	18.2	18.9	20.6	22.3
Liver cancer	5.6	5.4	6.8	6.7	9.7	9.8	11.5	10.9	14.3	14.9	21.0	20.3
Metabolic	3.1	3.9	2.7	2.8	2.0	2.7	3.5	3.7	3.3	2.9	2.5	2.8
Fulminant	10.9	11.0	9.7	12.1	8.4	9.1	4.8	4.2	4.5	3.2	3.0	2.3
Autoimmune	32.3	29.4	29.6	24.4	25.6	22.3	11.1	10.7	8.7	8.7	7.6	6.3
Hepatitis C	18.9	19.5	19.3	20.9	18.8	18.6	34.0	34.0	33.7	34.1	27.9	27.9
Others	19.0	20.4	20.6	21.3	22.1	24.6	16.7	17.3	16.6	16.7	16.9	17.7
Cold ischemia time, h (%)												
<8	50.0	50.9	52.6	55.6	58.2	57.8	54.1	51.8	52.8	53.8	57.4	54.9
8–<12	32.2	32.0	33.2	31.7	32.4	32.5	32.0	32.4	34.0	33.3	32.6	34.9
≥12	17.8	17.1	14.2	12.7	9.4	9.8	13.9	15.8	13.1	12.9	9.9	10.3
Missing	13.9	13.2	13.1	12.6	13.9	12.9	12.5	12.1	11.9	13.0	14.9	14.5
Transplant year (%)												
1988–1994	24.2	25.2	12.4	11.2	4.1	4.3	13.8	17.8	7.5	7.3	2.2	2.3
1995–1999	21.5	23.3	20.8	19.8	14.4	13.0	20.5	21.0	16.4	15.8	10.7	10.7
2000–2004	21.5	19.2	23.9	21.1	22.0	22.5	23.1	22.4	23.8	24.2	23.3	22.9
2005–2009	16.7	16.5	22.0	22.9	28.4	27.6	20.9	20.1	26.6	26.3	30.7	30.8
2010–2014	11.4	11.0	15.1	17.9	22.9	23.8	15.2	13.2	19.2	18.8	24.1	24.1
2015–2019	4.6	4.7	5.8	7.0	8.2	8.8	6.3	5.5	6.5	7.5	8.9	9.2
Donor:recipient weight ratio^*a*^ (%)												
≥0.9	55.7	68.1	62.8	73.8	60.1	75.7	39.6	55.0	45.9	64.6	44.2	62.1
<0.9	44.3	31.9	37.2	26.2	39.9	24.3	60.4	45.0	54.1	35.4	55.8	37.9
Missing	8.8	7.8	5.7	4.4	4.0	2.0	5.1	6.2	3.6	3.8	2.4	2.7
Graft size (%)												
Whole	95.7	94.3	98.7	98.2	99.8	99.8	97.7	97.3	99.1	99.3	99.8	99.7
Reduced	4.3	5.7	1.3	1.8	0.2	0.2	2.3	2.7	0.9	0.7	0.2	0.3
Donor CMV												
Positive	59.2	51.4	67.2	60.0	76.9	69.1	62.4	53.2	68.9	59.0	73.5	67.4
Negative	40.8	48.6	32.8	40.0	23.1	30.9	37.6	46.8	31.1	41.0	26.5	32.6
Missing	5.3	5.1	6.4	6.5	14.5	13.3	6.5	6.3	9.4	10.0	24.1	24.0
Donor hypertension (%)												
No	89.6	94.5	67.3	64.6	56.1	52.6	86.2	91.9	62.5	63.6	59.3	57.6
Yes	10.4	5.5	32.7	35.4	43.9	47.4	13.8	8.1	37.5	36.4	40.7	42.4
Missing	17.7	18.7	9.8	7.9	4.6	4.5	10.5	14.1	6.5	6.4	3.1	3.3
Donor cause of death (%)												
Cerebrovascular accident	61.7	72.7	47.5	51.6	45.6	46.8	62.9	71.7	46.4	51.2	51.9	51.5
Other	38.3	27.3	52.5	48.4	54.4	53.2	37.1	28.3	53.6	48.8	48.1	48.5
Missing	3.6	3.2	2.6	2.7	2.1	2.1	2.9	2.9	2.5	2.2	2.0	2.2
Donation after circulatory death (%)												
No	98.8	98.6	99.1	98.5	99.8	99.5	98.4	98.0	98.7	98.4	99.7	99.4
Yes	1.2	1.4	0.9	1.5	0.2	0.5	1.6	2.0	1.3	1.6	0.3	0.6
Missing	3.0	2.8	1.9	2.1	0.7	1.0	2.3	2.4	1.7	1.3	0.8	0.9

Because the unit of analysis was person-time, rather than person, the characteristics presented are weighted by a factor derived from the number of person-years of observation and number of events. For example, 2.3% of the person-years contributed by female recipients of female donors between 13 and 44 y were from people with congenital liver disease/biliary atresia as the primary disease.

^*a*^SRTR only.

CMV, cytomegalovirus; CTS, Collaborative Transplant Study; SRTR, Scientific Registry of Transplant Recipients.

Model for End-stage Liver Disease (MELD) scores were largely missing before 2003. After 2003, MELD scores were available for virtually all SRTR patients but only 34% of CTS patients. **Table S5** (**SDC,** https://links.lww.com/TXD/A830) compares MELD scores between the SRTR and CTS cohorts by donor-recipient sex combination. Acknowledging that MELD scores were missing for 66% of recipients in the CTS, recipients in the CTS appeared to have higher MELD scores than recipients in SRTR.

### Comparison of Mortality Rates by Donor Sex

**FIGURE 1.**
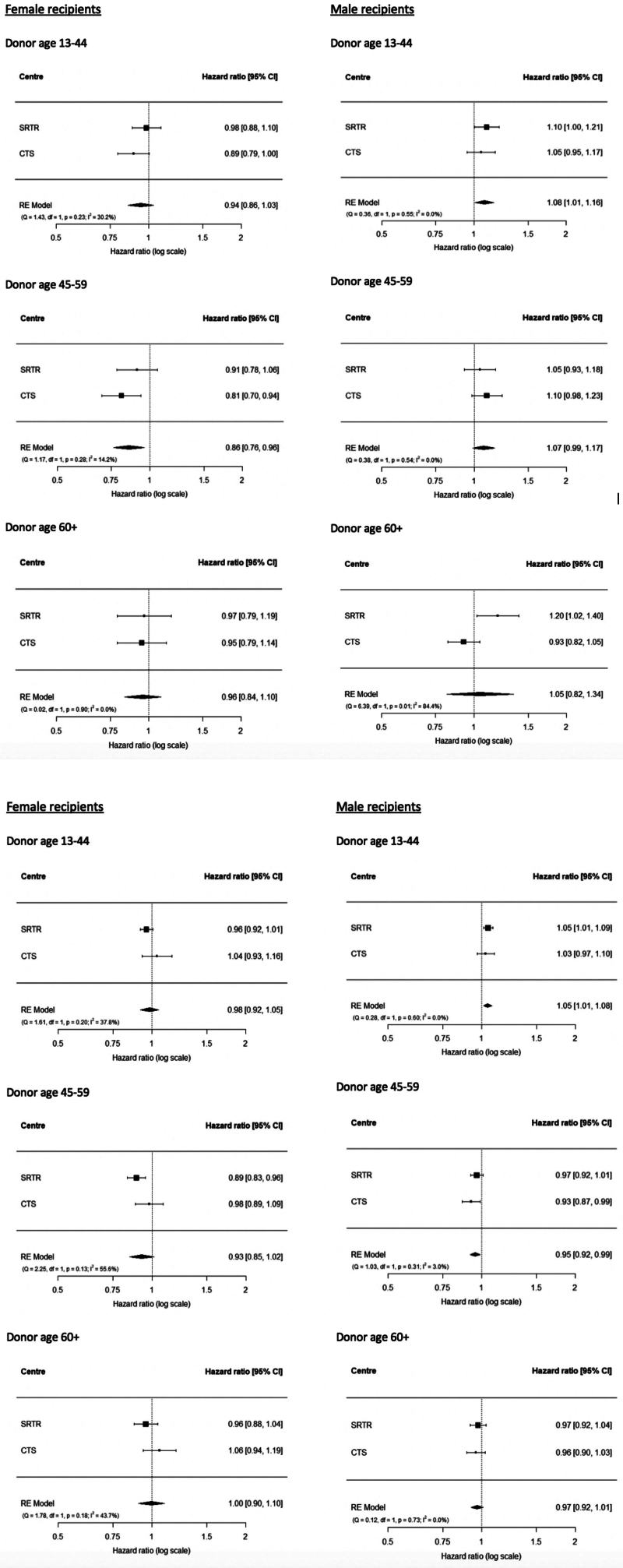
Association between donor sex and mortality in the first 3 mo posttransplant (A) and time subsequent to 3 mo (B) for female and male recipients. Forest plots show the adjusted HR for mortality in female and male recipients of female relative to male donors of each donor age. HR are shown separately for the SRTR and CTS cohorts, as well as the pooled HR from the meta-analysis. Models were adjusted for recipient race, primary disease (hepatitis C, acute fulminant liver failure, cancer, alcoholic liver disease, autoimmune, metabolic, congenital, other), cold ischemia (continuous linear), transplant year (continuous linear), reduced liver vs whole, donor-recipient weight ratio (≥0.9 vs <0.9, SRTR only), donor hypertension, donor CMV, donor CVA as cause of death, and age at transplant (piecewise linear). CTS, Collaborative Transplant Study; CVA, cerebrovascular accident; HR, hazard ratio; SRTR, Scientific Registry of Transplant Recipients.

**FIGURE 2.**
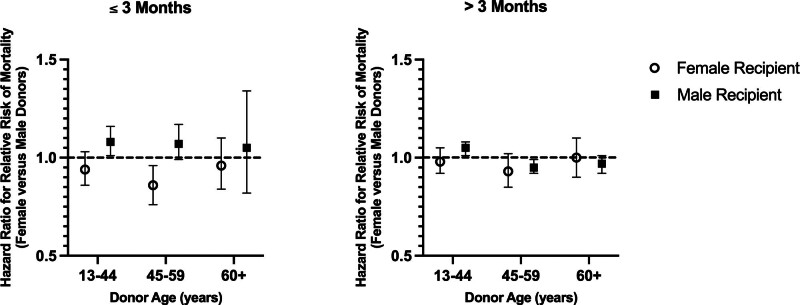
Summary of multivariable pooled hazard ratios for mortality in female and male recipients of female relative to male donor livers in the first 3 mo posttransplant and after the first 3 mo posttransplant. Models were adjusted for age at transplant, recipient race, primary disease, donor age, cold ischemia, transplant year, reduced liver vs whole, donor-recipient weight ratio (SRTR only), donor hypertension, donor CMV, and donor CVA as cause of death. CMV, cytomegalovirus; CVA, cerebrovascular accident; SRTR, Scientific Registry of Transplant Recipients.

Figure 1 shows forest plots indicating the adjusted relative hazards of death among female and male recipients of female compared with male donors in the first 3 mo posttransplant (short term) and after the first 3 mo posttransplant (long term). Pooled aHRs are shown as well as cohort-specific aHRs. Unadjusted HRs are shown in **Figure S1A and B** (**SDC,** https://links.lww.com/TXD/A830). Figure 2 summarizes the pooled adjusted HR for female versus male donors among female and male recipients within the 2-time intervals.

#### Female Recipients

In the first 3 mo posttransplant, among female recipients, the hazard of mortality was lower with a female donor than male donor regardless of donor age, but the difference was only statistically significant when the donor was aged 45–59 y (aHR 0.86 [95% CI, 0.76-0.96]). After the first 3 mo, among recipients of donors 45–59 y, mortality was lower with female than male donors (aHR 0.93 [95% CI, 0.85-1.02]), but the difference was not statistically significant. There were no differences in mortality by donor sex after the first 3 mo for recipients of donors aged 13–44 y or 60 y or older.

#### Male Recipients

Among male recipients, within the first 3 mo posttransplant, mortality was higher with female than male donors of all ages, but the difference was only statistically significant when the donor was 13–44 y (HR 1.08, [95% CI, 1.01-1.16]). After the first 3 mo, male recipients showed significantly higher mortality with female than male donors 13–44 y old (HR 1.05 [95% CI, 1.01-1.09]), but lower mortality with female than male donors 45 y or older, although the difference was only statistically significant for donors 45–59 y (HR 0.95, [95% CI, 0.92-0.99]).

### Between-cohort Heterogeneity

There was some heterogeneity in outcomes between SRTR and CTS, but CIs were still largely overlapping between the 2 cohorts. In first 3 mo, the greatest heterogeneity between SRTR and CTS was seen among female recipients of donors 13–44 y (*I*^*2*^ = 30.2%) and among male recipients of donors aged 60 y or older (*I*^*2*^ = 84.4%). After the first 3 mo, there was some heterogeneity between SRTR and CTS among female recipients of donors of all ages (13–44 y, *I*^*2*^ = 37.8%; 45–59 y, *I*^*2*^ = 55.6%; 60 y or older, *I*^*2*^ = 43.7%); there was no heterogeneity in male recipients of any donor age. It is unclear whether these represent true differences between cohorts or random variation.

### Sensitivity Analysis

Because the donor-recipient weight ratio was available only for SRTR, the model was refitted excluding this variable to determine whether this may have contributed to between-cohort heterogeneity. Results of a sensitivity analysis that adjusted for donor-recipient weight ratio (**Table S6, SDC,** https://links.lww.com/TXD/A830) were almost identical to those of the primary analysis.

### Comparison of Graft Failure by Donor Sex

The results of analyses comparing graft failure rates by donor sex in the SRTR cohort are shown in **Figure S2** (**SDC,** https://links.lww.com/TXD/A830).

#### Female Recipients

In the first 3 mo posttransplant, graft failure rates were lower with female than male donors across all ages, but these differences were not statistically significant. After the first 3 mo, graft failure rates were significantly lower with female than male donors 45–49 y (aHR 0.82 [0.72-0.93]) and 60 y or older (aHR 0.81 [0.70-0.94]).

#### Male Recipients

In the first 3 mo posttransplant, graft failure rates were significantly higher with female than male donors of all ages (13–44 y: aHR 1.39 [1.23-1.57]; 45–59 y: aHR 1.32 [1.15-1.51]; 60 y or older: aHR 1.22 [1.03-1.43]). After the first 3 mo, graft failure rates were nonsignificantly higher with female than male donors 13–44 y (aHR 1.07 [0.99-1.16]) but *lower* with female than male donors 45–59 y (aHR 0.91 [0.84-0.99]) and 60 y or older (aHR 0.92 [0.83-1.03]); the difference was only statistically significant with donors 45–59 y.

Overall, the HRs for the relationship between donor sex and graft failure were similar in direction but larger in magnitude compared with those for the relationship between donor sex and mortality.

### Crude Absolute Mortality Rates

**FIGURE 3.**
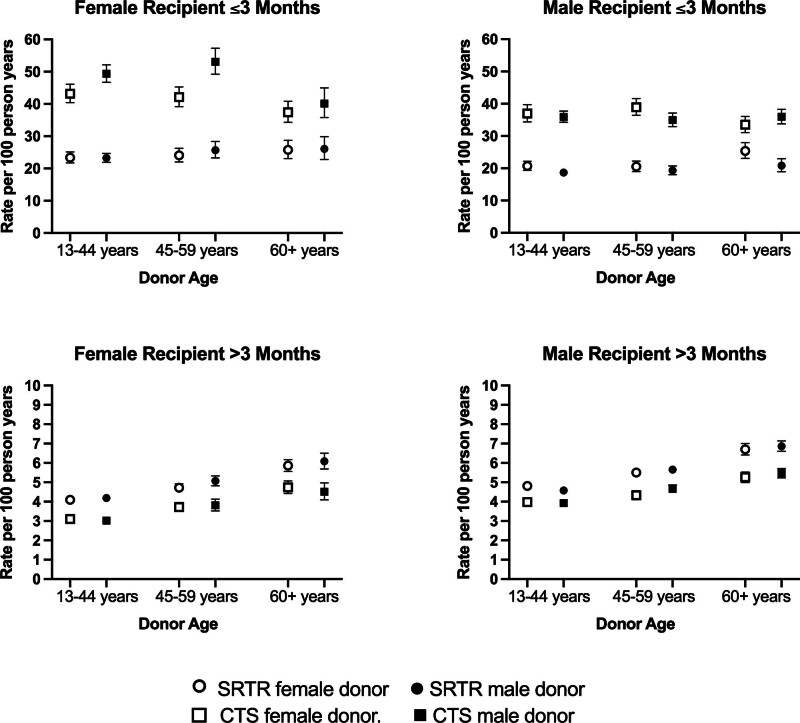
Crude mortality rates for female and male recipients of female and male donors in the first 3 mo and after the first 3 mo posttransplant by donor sex and age. Rates are shown for SRTR and CTS separately. CTS, Collaborative Transplant Study; SRTR, Scientific Registry of Transplant Recipients.

Crude mortality rates were much lower in the SRTR than the CTS within the first 3 mo posttransplant. In contrast, beyond the first 3 mo, crude mortality rates for CTS were slightly lower than for SRTR. Absolute differences in crude mortality rates by donor sex were small. As shown in Figure 3, the absolute differences in crude mortality rates by donor sex ranged from 0.09 to 11.0 deaths per 100 person-years in the first 3 mo posttransplant and from 0.08 to 0.33 deaths per 100 person-years in the time after the first 3 mo.

## DISCUSSION

Estimation of the association between donor sex and mortality among liver transplant recipients from 2 of the largest transplant registries worldwide showed few substantial differences in mortality by donor sex. Male recipients showed slightly higher early posttransplant mortality with female than male donors of all ages, although only significantly with the youngest donors. In the long-term, among male recipients, mortality was higher with female than male donors 13–44 y but *lower* with female than male donors aged 45 y or older. Female recipients of donors aged 45–59 y showed slightly lower short-term and long-term mortality with female donors than with male donors, although this was only statistically significant in the early posttransplant period.

Any differences in patient mortality by donor sex are almost certainly driven by donor sex-related differences in graft survival. It is difficult to imagine how donor sex could directly influence patient mortality from causes other than graft failure. Comparisons of mortality by donor sex will always be limited by “noise” introduced by deaths unrelated to graft failure.

Our analysis, limited to the SRTR, of the association between donor sex and graft survival showed a similar pattern to that observed in kidney transplant.^24^ In the interval after the first 3 mo, both female and male liver recipients showed lower graft failure rates with female than male donors aged 45 y or older; in kidney transplant, both female and male recipients showed better graft survival with female than male donors aged 60 y or older.^24^ The lower graft failure rates observed among female recipients of older female than male donors may be partly related to an immune reaction against the HY antigen present on all male tissues.^25,26^ However, if this were the only explanation, 1 would expect similarly lower graft failure rates with female than male younger donors as well, which was not the case. The fact that male recipients of donors 45 y or older also showed lower graft failure rates and mortality with a female than a male donor after the first 3 mo posttransplant suggests that other nonimmunologic factors may be at play. Female kidneys appear to age more slowly than male kidneys^27^; perhaps the same is true for livers. A greater functional reserve, or regenerative capacity, in older female than male livers should also be considered.

The higher mortality observed among male recipients of young female than male donors raises the possibility that differences between young male and female donors in expression of sex hormone receptors on liver tissue may result in differential adaptation of the grafted organ to the recipient environment.^10,28^ Some have suggested that an “abrupt defeminization” of livers from female donors transplanted into male recipients,^10^ resulting in rapid reduction in estrogen receptor activity, may result in greater ischemia/reperfusion injury and poorer regenerative capacity. This was supported by a recent study that evaluated the presence of estrogen receptors on liver tissue biopsies at the time of donor hepatectomy. This study suggested that an acute decrease in hepatic estrogen signaling when a liver from a female donor younger than 40 y is transplanted into a male recipient may result in liver injury due to greater ischemia/reperfusion injury poorer regenerative capacity and thereby contribute to the higher graft failure rates observed among male recipients of female donors younger than 40 y compared with all other donor-recipient sex combinations when the donor is younger than 40 y.^28^ Interestingly, male recipients of female donors younger than 40 y only showed poorer outcomes than other donor-recipient sex combinations when macrosteatosis (a measure of donor quality) was absent.^28^ Livers from female donors of postreproductive age, in which estrogen receptor activity is already reduced, may behave more similarly to livers from younger male donors. Interestingly, our study showed that male recipients had higher early posttransplant mortality associated with female than male donors of all ages. Although this was statistically significant only among the youngest donors, failure to detect significant mortality differences at older donor ages may reflect inadequate power to detect differences in these older donor age categories. Early posttransplant graft failure rates among male recipients were significantly higher with female than male donors of all ages.

Another notable finding of the present study is the important differences between the CTS and SRTR cohorts in crude absolute mortality rates. In the first 3 mo posttransplant, crude mortality rates were substantially higher in the CTS than the SRTR. This likely reflects differences in allocation policies between the United States and the countries contributing to CTS. Based on the available MELD scores, the CTS cohort appears to have been sicker at the time of transplant than the SRTR cohort. Additionally, cold ischemia times were longer in the CTS compared with the SRTR which may have resulted in more ischemia/reperfusion injury leading to primary nonfunction, early allograft dysfunction, rejection and overall decreased graft and patient survival.^29-31^ In contrast, crude absolute mortality rates were slightly lower in the CTS than the SRTR in the period subsequent to the first 3 mo. This may reflect selection bias whereby only the healthiest patients in the CTS survived the first 3 mo. This may also reflect differences in baseline mortality risk between the United States (SRTR) and the countries represented in the CTS due to myriad social and other factors.^32,33^ We must also consider the possibility that differences in donor selection, management strategies, and healthcare insurance policies between the United States and Europe played a role, as has been observed in kidney transplantation.^34^ More work is needed to understand the factors that may contribute to the observed differences.

It is important to recognize that in both the SRTR and CTS, the absolute differences in crude mortality rates by donor sex were very small and would not justify any change in organ allocation or acceptance practices. Nevertheless, these observations, considered in the context of differences in graft outcomes by donor sex in other organ types, helps enhance our understanding of the factors influencing graft and patient survival.

This was the largest study to date assessing the association between donor sex and graft outcomes; the combination of 2 of the largest existing transplant databases worldwide is an important strength. However, there are several limitations. Importantly, death due to graft loss could not be distinguished from death with graft function in the CTS; therefore, to pool analyses across the 2 cohorts, we used mortality as the primary outcome. Although graft survival is the most important determinant of patient survival, some patients die with graft function. We expect that the relationship between donor sex and mortality is determined primarily by its association with graft survival; if there is no association between donor sex and death with graft function (which is likely), using mortality as the outcome will lead to HR estimates that are biased toward no difference by donor sex and wider CIs compared with analyses with an outcome of graft loss (defined as retransplant or death due to graft failure). Residual confounding is also a possibility. Measures of organ quality that could differ by donor sex were not available. Donor sex is unlikely to be associated with specific recipient factors other than recipient sex; therefore, we believe that major confounding by recipient factors is unlikely. Additionally, donor:recipient weight ratio was included in models for the SRTR cohort but was not available for the CTS cohort. The results of models in SRTR excluding this variable were virtually identical to those including donor:recipient weight ratio, suggesting that it was not functioning as a confounder. Therefore, it is unlikely that inclusion of this variable in 1 cohort but not the other had an impact on heterogeneity between the cohorts. It is not possible to determine the factors contributing to the observed heterogeneity between SRTR and CTS. Finally, this study cannot identify the reasons for the observed differences in mortality by donor sex.

## CONCLUSIONS

By combining data from the 2 largest transplant databases worldwide, we show a modifying effect of donor age on the association between donor sex and recipient survival. Among male recipients, early posttransplant mortality was higher with female than male donors, whereas after the first 3 mo, young female donors were associated with higher mortality than young male donors, whereas female donors aged 45 y or older were associated with lower mortality than same-aged male donors. These finding reinforce the need to consider biological sex in the context of age and suggest that older female liver donors may offer some advantages over older male liver donors. We also observed substantially higher early posttransplant mortality in the Europe-focused CTS than in the SRTR. Additional studies are needed to determine the mechanisms underlying our observations and the reasons for the differences in early mortality in the 2 databases.

## ACKNOWLEDGMENTS

The authors thank the transplant registries Eurotransplant, Italian Centro Nazionale Trapianti, Organització Catalana de Trasplantaments, Nederlandse Transplantatie Stichting, and UK Transplant for collaboration and data exchange with CTS and gratefully acknowledge the generous support of the transplantation centers or hospitals that provided data for this study to CTS.

## Supplementary Material


