# Supplementary material for: Differences in Mortality by Donor Sex and Age in a Multinational Cohort of Liver Transplant Recipients
Source: Transplant Direct. 2026 Feb 11;12(3):e1912. doi: 10.1097/TXD.0000000000001912 (PMC12900217; doi:10.1097/TXD.0000000000001912)
Supplement: Supplementary file 1 [file txd-12-e1912-s001.pdf]

Supplemental Data

Countries included in the CTS dataset for this study:

This study included data from centers in Australia, Austria, Belgium, Canada, Columbia, France, Germany, Hungary, Ireland, Israel, Italy, Netherlands, Slovenia, Spain, Sweden, Switzerland, Turkey, and the United Kingdom. To avoid duplicates with the SRTR, recipients from USA were excluded from the CTS cohort.

Table S1: Composition of the contrasted experience of SRTR patients, by donor sex and donor age, for female and male recipients within first 3 months post-transplant (proportion per 100 patient years)

	Female Recipients						Male Recipients					
**Donor Age**	**13-44 years**		**45-59 years**		**60+ years**		**13-44 years**		**45-59 years**		**60+ years**	
**Donor sex**	**Female**	**Male**	**Female**	**Male**	**Female**	**Male**	**Female**	**Male**	**Female**	**Male**	**Female**	**Male**
**# Patients**	10271	15501	6783	4874	4027	2534	13968	33234	10239	13808	5285	6594
**Person-years of observation**	2402	3623	1571	1130	928	587	3274	7872	2388	3258	1213	1550
**Deaths**	561	843	378	290	239	153	679	1470	490	629	308	323
**Death/100 pyrs**	23.4	23.3	24.1	25.7	25.8	26.1	20.7	18.7	20.5	19.3	25.4	20.8
**Age at Transplant (%)**												
13-<20y	4.0	4.3	1.4	1.2	0.6	0.5	2.0	1.4	0.7	0.3	0.3	0.1
20- <35y	9.2	9.4	6.9	6.8	3.8	4.6	4.7	4.4	3.6	2.9	2.4	1.6
35+y	86.9	86.3	91.7	91.9	95.5	94.8	92.5	93.3	95.0	96.3	96.9	97.8
**Recipient race (%)**												
White	83.2	84.2	84.2	84.2	85.8	86.5	86.3	88.3	86.8	88.7	85.6	88.9
Black	10.8	11.0	10.5	10.8	8.0	8.4	7.7	7.1	7.3	6.8	7.4	6.2
Others	6.0	4.7	5.3	5.0	6.2	5.2	6.0	4.6	6.0	4.5	7.1	5.0
Missing												
**Primary disease (%)**												
Congenital/Biliary atresia	1.6	1.5	0.8	0.8	0.6	0.5	0.9	0.8	0.5	0.4	0.3	0.3
Alcohol	10.4	10.8	11.6	12.0	11.3	11.6	18.8	19.4	18.6	18.8	21.1	22.0
Liver Cancer	8.9	8.0	10.0	10.1	12.9	12.6	15.7	14.6	18.1	18.3	21.5	20.3
Metabolic	2.6	3.1	2.2	2.2	1.6	2.6	2.9	2.9	2.7	2.4	2.4	2.7
Fulminant	8.6	9.5	6.7	8.7	5.8	5.8	3.8	3.7	3.6	2.9	2.8	2.4
Autoimmune	24.5	23.0	21.2	19.0	22.0	18.5	8.5	8.6	6.9	6.6	7.4	6.3
Hepatitis C	21.8	21.4	23.5	23.8	18.4	19.3	32.9	32.7	32.9	33.7	25.2	26.6
Others	21.7	22.8	24.0	23.3	27.4	29.1	16.6	17.3	16.7	17.0	19.4	19.5
**Cold ischemia time (%)**												
<8h	62.5	62.3	66.4	68.8	70.4	69.6	65.0	62.2	65.4	66.7	68.6	67.9
8-<12h	26.6	26.9	25.8	24.8	24.7	25.2	26.6	27.5	27.4	26.5	25.7	26.3
>=12h	10.9	10.8	7.8	6.4	5.0	5.3	8.4	10.2	7.2	6.8	5.7	5.8
Missing	6.0	6.3	5.1	4.6	5.0	4.4	5.8	5.9	4.9	4.5	4.4	4.2
**Transplant year (%)**												
1988-1994	15.8	16.8	9.1	6.4	4.0	3.5	9.1	12.6	5.2	5.1	2.7	2.6
1995-1999	14.3	15.0	12.6	11.2	11.6	9.6	12.5	12.8	10.3	9.1	9.1	8.8

2000-2004	14.9	14.2	16.2	12.7	15.9	15.9	15.5	15.7	16.0	15.3	17.3	16.3
2005-2009	15.2	15.8	19.3	19.9	22.2	20.8	18.0	18.0	21.9	22.0	22.7	23.3
2010-2014	17.3	16.6	19.3	22.8	20.8	22.4	20.0	18.1	23.1	21.9	22.3	22.0
2015-2019	22.5	21.6	23.6	27.1	25.6	27.9	24.9	22.8	23.5	26.7	26.0	27.1
**Donor:recipient weight ratio* (%)**												
≥0.9	56.9	68.4	61.9	72.8	59.5	73.4	42.3	56.3	46.7	64.7	44.3	61.8
<0.9	43.1	31.6	38.1	27.2	40.5	26.6	57.7	43.8	53.3	35.3	55.8	38.3
Missing	5.8	5.9	4.2	2.7	3.0	2.0	4.0	4.6	2.7	2.8	2.1	2.4
**Graft size (%)**												
Whole	97.2	95.9	99.6	99.7	100.0	100.0	98.8	98.4	99.9	99.9	99.9	100.0
Reduced	2.8	4.1	0.4	0.4	0.0	0.1	1.2	1.6	0.1	0.1	0.1	0.1
**Donor CMV (%)**												
Positive	64.6	55.9	71.8	63.7	78.9	72.8	66.0	56.0	72.0	60.9	76.7	70.4
Negative	35.4	44.1	28.3	36.3	21.1	27.2	34.0	44.0	28.0	39.2	23.3	29.6
Missing	0.7	0.9	0.7	0.6	0.5	0.6	0.7	0.7	0.4	0.5	0.3	0.5
**Donor Hypertension (%)**												
No	83.2	90.9	50.6	48.7	34.5	32.9	80.5	87.8	46.6	46.6	32.8	31.2
Yes	16.9	9.1	49.4	51.3	65.5	67.1	19.5	12.2	53.4	53.4	67.3	68.8
Missing	14.1	15.1	8.4	6.0	3.9	3.8	8.2	11.3	4.9	5.3	2.7	3.1
**Donor cause of death (%)**												
Cerebrovascular accident	68.8	87.6	32.5	45.7	24.2	35.4	69.0	85.4	34.8	47.5	25.7	35.0
Other	31.2	12.5	67.5	54.3	75.8	64.6	31.0	14.6	65.2	52.5	74.3	65.0
Missing	0.1	0.1	0.1	0.0	0.1	0.0	0.1	0.1	0.1	0.1	0.0	0.1
**Donation after circulatory death (%)**												
No	96.8	96.7	97.6	96.3	99.7	99.6	96.2	95.4	97.4	96.3	99.8	99.5
Yes	3.2	3.3	2.4	3.7	0.4	0.5	3.8	4.6	2.6	3.7	0.3	0.5

Because the unit of analysis was person-time, rather than person, the characteristics presented are weighted by a factor derived from the number of person-years of observation and number of events. For example, 1.6% of the person-years contributed by female recipients of female donors between 13 and 44 years were from people with congenital liver disease/ biliary atresia as the primary disease.

CMV: cytomegalovirus

*****Donor:recipient weight ratio was available only for the SRTR cohort

Table S2: Composition of the contrasted experience of SRTR patients, by donor sex and donor age, for female and male recipients after first 3 months post-transplant (proportion per 100 patient years)

	Female Recipients						Male Recipients					
**Donor Age**	**13-44 years**		**45-59 years**		**60+ years**		**13-44 years**		**45-59 years**		**60+ years**	
**Donor sex**	**Female**	**Male**	**Female**	**Male**	**Female**	**Male**	**Female**	**Male**	**Female**	**Male**	**Female**	**Male**
**# Patients**	9294	14045	6076	4363	3560	2250	12674	30659	9225	12640	4650	5960
**Person-years of observation**	75576	114724	44705	28744	22917	13409	89769	233147	60131	79603	27765	34202
**Deaths**	3098	4809	2110	1457	1342	816	4316	10681	3307	4500	1860	2347
**Death/100 pyrs**	4.1	4.2	4.7	5.1	5.9	6.1	4.8	4.6	5.5	5.7	6.7	6.9
**Age at Transplant (%)**												
13-<20y	4.0	4.3	1.6	1.4	0.8	0.6	2.4	1.7	1.0	0.5	0.6	0.1
20- <35y	10.1	10.0	7.8	7.1	4.7	5.1	5.7	5.6	4.6	3.8	3.3	2.1
35+y	86.0	85.7	90.6	91.5	94.5	94.3	91.9	92.7	94.4	95.7	96.1	97.8
**Recipient race (%)**												
White	84.6	85.6	85.1	85.2	86.7	87.0	86.9	89.4	86.8	89.3	85.1	89.6
Black	9.9	10.1	9.7	9.8	7.4	7.9	6.9	6.4	6.5	6.1	6.7	5.2
Others	5.6	4.3	5.2	5.1	6.0	5.2	6.2	4.2	6.6	4.6	8.2	5.2
Missing												
**Primary disease (%)**												
Congenital/Biliary atresia	1.7	1.5	0.9	0.7	0.8	0.9	1.1	0.9	0.6	0.3	0.5	0.3
Alcohol	7.8	8.6	9.7	9.8	10.6	9.8	17.3	18.0	17.1	17.9	22.4	22.6
Liver Cancer	6.4	5.7	7.5	7.6	9.2	9.3	11.7	10.5	15.0	14.7	18.0	16.8
Metabolic	2.9	3.5	2.5	2.5	1.9	2.7	3.2	3.5	3.3	2.9	3.0	3.5
Fulminant	9.8	10.0	7.8	10.3	7.7	8.2	4.3	4.2	4.3	3.2	3.1	2.7
Autoimmune	31.2	29.0	27.2	23.8	27.3	23.8	10.6	10.8	8.3	8.6	8.6	7.7
Hepatitis C	19.9	20.1	21.0	21.8	15.6	16.0	34.9	34.5	34.0	35.4	24.4	25.2
Others												
**Cold ischemia time (%)**												
<8h	54.0	54.6	59.1	60.9	63.8	63.0	58.2	54.8	58.9	60.1	64.0	60.8
8-<12h	30.9	31.0	30.5	30.1	29.8	30.4	30.9	31.7	31.9	31.0	29.1	31.5
>=12h	15.1	14.4	10.4	9.0	6.4	6.6	10.9	13.5	9.2	9.0	7.0	7.7
Missing	7.7	8.1	7.0	6.5	7.0	5.6	7.8	7.5	6.4	6.3	6.1	5.0
**Transplant year (%)**												
1988-1994	24.2	24.6	13.4	9.8	5.7	5.6	12.4	17.7	7.0	7.3	3.4	3.5

1995-1999	19.7	21.7	19.0	18.2	16.7	13.5	18.8	19.1	15.0	14.0	12.8	12.8
2000-2004	20.0	18.3	22.5	18.2	22.2	21.9	22.4	21.4	22.3	22.2	24.3	22.8
2005-2009	17.1	17.0	22.1	24.9	28.2	27.3	21.7	20.8	27.0	27.3	28.6	29.2
2010-2014	13.2	12.5	16.0	20.1	19.0	22.3	17.1	14.5	20.9	20.3	21.5	22.2
2015-2019	5.9	5.8	7.0	8.7	8.2	9.4	7.7	6.5	7.9	8.9	9.4	9.6
**Donor:recipient weight ratio* (%)**												
≥0.9	55.8	68.1	62.8	73.8	60.1	75.7	39.7	55.0	45.9	64.6	44.2	62.1
<0.9	44.3	31.9	37.2	26.2	39.9	24.4	60.4	45.0	54.2	35.4	55.8	37.9
Missing	8.8	7.8	5.7	4.4	4.0	2.0	5.2	6.2	3.6	3.8	2.4	2.7
**Graft size (%)**												
Whole	97.5	96.2	99.5	99.6	99.9	100.0	98.8	98.6	99.8	99.8	99.9	99.9
Reduced	2.5	3.8	0.5	0.4	0.1	0.0	1.2	1.4	0.2	0.2	0.1	0.1
**Donor CMV (%)**												
Positive	62.3	53.6	69.9	63.1	79.6	73.2	65.2	55.4	71.5	60.8	76.0	70.6
Negative	37.7	46.4	30.1	36.9	20.4	26.8	34.8	44.6	28.5	39.3	24.1	29.4
Missing	0.8	1.0	0.7	0.6	0.7	0.6	0.8	0.7	0.4	0.6	0.4	0.5
**Donor Hypertension (%)**												
No	85.3	92.7	53.4	51.2	36.6	34.4	82.6	89.7	49.2	49.2	36.0	32.3
Yes	14.7	7.3	46.6	48.8	63.4	65.6	17.4	10.3	50.8	50.8	64.0	67.7
Missing	21.4	21.6	12.1	8.4	5.2	5.0	10.9	15.4	6.6	7.0	3.4	3.6
**Donor cause of death (%)**												
Cerebrovascular accident	65.7	87.5	27.3	43.2	20.5	31.4	66.4	85.2	29.2	42.9	23.2	30.9
Other	34.3	12.5	72.7	56.9	79.5	68.6	33.6	14.8	70.8	57.1	76.9	69.1
Missing	0.1	0.1	0.1	0.1	0.1	0.0	0.2	0.1	0.1	0.1	0.0	0.1
**Donation after circulatory death (%)**												
No	98.4	98.4	98.9	98.2	99.9	99.8	98.0	97.6	98.5	98.1	99.9	99.9
Yes	1.6	1.7	1.1	1.8	0.1	0.2	2.0	2.4	1.5	1.9	0.1	0.1

Because the unit of analysis was person-time, rather than person, the characteristics presented are weighted by a factor derived from the number of person-years of observation and number of events. For example, 1.7% of the person-years contributed by female recipients of female donors between 13 and 44 years were from people with congenital liver disease/ biliary atresia as the primary disease.

CMV: cytomegalovirus

*Donor:recipient weight ratio was available only for the SRTR cohort

Table S3: Composition of the contrasted experience of CTS patients, by donor sex and donor age, for female and male recipients within first 3 months post-transplant (proportion per 100 patient years)

	Female Recipients						Male Recipients					
**Donor Age**	**13-44 years**		**45-59 years**		**60+ years**		**13-44 years**		**45-59 years**		**60+ years**	
**Donor sex**	**Female**	**Male**	**Female**	**Male**	**Female**	**Male**	**Female**	**Male**	**Female**	**Male**	**Female**	**Male**
**# Patients**	5226	6049	4606	2727	3789	1984	5606	13035	6098	8703	6054	7861
**Person-years of observation**	1151.1	1324.9	1013.9	589.7	846.9	441.2	1257.8	2954.7	1361.2	1964.0	1367.3	1782.4
**Deaths**	497	654	427	313	317	177	465	1062	530	687	458	641
**Death/100 pyrs**	43.2	49.4	42.1	53.1	37.4	40.1	37.0	35.9	38.9	35.0	33.5	36.0
**Age at Transplant (%)**												
13-<20y	4.8	5.1	3.3	3.2	0.9	0.9	3.8	2.2	1.9	0.8	0.3	0.2
20- <35y	15.1	14.8	11.4	11.5	6.6	5.8	9.5	8.7	6.9	5.5	3.0	2.6
35+y	80.1	80.1	85.3	85.4	92.5	93.4	86.7	89.1	91.2	93.6	96.7	97.2
**Recipient race (%)**												
White	92.9	93.0	94.4	93.9	95.3	95.8	92.5	93.1	93.8	95.1	94.7	96.4
Black	1.7	1.2	1.5	1.7	1.7	2.2	1.7	0.9	1.5	1.2	1.2	1.1
Others	5.4	5.8	4.1	4.4	3.0	2.0	5.8	5.9	4.7	3.7	4.0	2.5
Missing	35.1	34.4	40.3	38.1	55.5	50.9	40.1	39.5	45.7	47.0	64.0	62.6
**Primary disease (%)**												
Congenital/Biliary atresia	3.6	4.0	3.4	4.8	2.8	4.5	1.4	1.0	1.1	0.8	0.6	0.5
Alcohol	8.9	9.2	10.7	11.0	14.5	13.5	17.4	19.3	20.4	21.3	20.5	22.0
Liver Cancer	6.0	6.0	7.6	6.3	11.9	12.1	13.5	14.5	15.1	17.7	24.5	24.4
Metabolic	3.6	4.5	3.2	3.5	2.4	3.3	3.6	3.5	3.4	2.8	2.1	2.3
Fulminant	12.4	13.0	12.4	14.8	8.2	9.5	5.7	4.2	4.8	3.2	2.9	2.2
Autoimmune	30.2	27.0	28.2	21.9	20.8	17.0	11.7	9.4	9.7	8.2	6.0	4.9
Hepatitis C	18.9	19.8	20.0	21.2	23.7	24.2	31.5	32.8	31.2	31.4	30.9	30.8
Others	16.3	16.6	14.5	16.4	15.7	16.0	15.1	15.3	14.2	14.6	12.5	12.9
**Cold ischemia time (%)**												
<8h	42.4	42.8	44.2	47.1	52.1	50.6	43.8	45.1	43.7	44.4	51.6	50.6
8-<12h	34.8	33.6	37.3	36.0	35.8	36.5	34.3	33.8	37.6	37.8	36.2	36.9
>=12h	22.8	23.6	18.5	16.9	12.1	12.8	21.9	21.1	18.7	17.9	12.2	12.5
Missing	26.7	26.5	26.1	25.4	26.3	25.7	25.0	26.1	24.4	26.3	27.1	26.3
**Transplant year (%)**												
1988-1994	18.5	22.1	7.8	9.7	1.1	1.5	13.6	15.1	6.4	5.9	0.8	0.8
1995-1999	19.4	21.5	15.7	16.5	6.8	7.9	19.7	20.3	13.9	13.1	5.3	5.7

2000-2004	20.9	19.8	20.1	21.4	13.7	15.4	20.5	20.6	19.7	19.4	13.9	14.5
2005-2009	17.9	15.6	20.9	18.2	22.1	21.6	18.6	18.0	22.8	21.4	24.3	23.5
2010-2014	12.8	11.8	20.2	19.1	30.4	29.0	16.0	14.7	21.7	21.4	30.2	29.1
2015-2019	10.5	9.2	15.3	15.1	25.9	24.7	11.5	11.3	15.5	18.8	25.4	26.5
**Graft size (%)**												
Whole	90.2	87.7	96.7	95.1	99.6	99.7	93.8	93.2	97.9	98.5	99.7	99.6
Reduced	9.8	12.3	3.3	4.9	0.4	0.3	6.2	6.8	2.1	1.5	0.3	0.4
**Donor CMV (%)**												
Positive	51.8	45.0	60.9	51.2	71.0	59.5	53.2	46.1	61.1	53.2	69.7	60.3
Negative	48.2	55.0	39.1	48.8	29.0	40.5	46.8	53.9	38.9	46.8	30.3	39.7
Missing	17.3	19.1	19.1	20.5	34.8	33.6	23.0	23.6	26.2	27.6	47.5	46.5
**Donor Hypertension (%)**												
No	96.5	98.2	88.1	88.1	80.8	78.4	95.1	97.4	86.5	87.0	81.5	81.1
Yes	3.5	1.8	11.9	11.9	19.2	21.6	4.9	2.6	13.5	13.0	18.5	18.9
Missing	11.0	11.4	6.6	7.2	3.7	3.8	10.2	11.0	7.1	6.0	3.4	3.1
**Donor cause of death (%)**												
Cerebrovascular accident	51.4	28.8	80.5	66.0	82.1	71.9	52.1	29.6	78.8	64.6	82.6	72.4
Other	48.6	71.2	19.5	34.0	17.9	28.1	47.9	70.4	21.2	35.4	17.4	27.6
Missing	11.5	11.6	7.2	7.8	4.7	5.4	10.5	11.2	7.8	6.7	4.6	4.2
**Donation after circulatory death (%)**												
No	99.3	99.1	98.8	98.8	99.3	98.6	98.7	98.6	98.5	98.0	99.1	98.5
Yes	0.7	0.9	1.2	1.2	0.7	1.4	1.3	1.4	1.5	2.0	0.9	1.5
Missing	9.5	10.4	5.4	5.8	2.2	2.8	8.6	9.5	5.6	4.4	2.1	1.9

Because the unit of analysis was person-time, rather than person, the characteristics presented are weighted by a factor derived from the number of person-years of observation and number of events. For example, 3.6% of the person-years contributed by female recipients of female donors between 13 and 44 years were from people with congenital liver disease/ biliary atresia as the primary disease.

CMV: cytomegalovirus

Table S4: Composition of the contrasted experience of CTS patients, by donor sex and donor age, for female and male recipients after first 3 months post-transplant (proportion per 100 patient years)

	Female Recipients						Male Recipients					
**Donor Age**	**13-44 years**		**45-59 years**		**60+ years**		**13-44 years**		**45-59 years**		**60+ years**	
**Donor sex**	**Female**	**Male**	**Female**	**Male**	**Female**	**Male**	**Female**	**Male**	**Female**	**Male**	**Female**	**Male**
**# Patients**	4375	5001	3826	2196	3159	1651	4789	11263	5125	7462	5149	6733
**Person-years of observation**	37601.1	43220.0	27009.5	15352.4	16484.1	8665.1	34779.7	84073.5	32845.0	45916.2	26446.1	33647.9
**Deaths**	1166	1304	1004	585	781	391	1384	3304	1423	2141	1390	1839
**Death/100 pyrs**	3.10	3.02	3.72	3.81	4.74	4.51	3.98	3.93	4.33	4.66	5.26	5.47
**Age at Transplant (%)**												
13-<20y	4.6	5.3	3.1	3.7	1.1	0.6	4.3	2.4	2.2	0.9	0.3	0.2
20- <35y	15.6	15.3	11.4	11.7	7.3	6.8	10.8	9.8	7.3	6.1	3.4	2.7
35+y	79.8	79.3	85.5	84.6	91.6	92.6	84.9	87.8	90.4	93.0	96.2	97.1
**Recipient race (%)**												
White	93.9	94.6	95.0	95.1	95.3	95.3	93.1	94.2	93.5	95.8	94.2	96.0
Black	1.5	0.9	1.3	1.2	1.4	2.7	1.4	1.0	1.2	1.0	1.4	1.2
Others	4.6	4.5	3.8	3.7	3.3	2.0	5.5	4.8	5.2	3.2	4.4	2.8
Missing	32.5	32.0	33.0	34.5	51.9	49.2	37.5	37.4	41.5	42.8	62.2	60.0
**Primary disease (%)**												
Congenital/Biliary atresia	3.4	3.9	2.9	4.8	2.6	4.1	1.5	0.9	1.0	0.8	0.5	0.5
Alcohol	8.1	7.7	9.4	9.7	13.5	12.5	16.9	18.9	20.1	20.8	18.8	22.0
Liver Cancer	4.0	4.5	5.8	5.1	10.4	10.6	11.0	11.8	12.9	15.3	24.0	23.8
Metabolic	3.7	4.7	3.1	3.3	2.2	2.6	4.1	4.3	3.5	3.0	2.1	2.2
Fulminant	13.0	13.6	12.7	15.4	9.5	10.4	5.9	4.3	4.9	3.3	3.0	1.9
Autoimmune	34.6	30.3	33.7	25.7	23.2	20.0	12.4	10.6	9.4	8.9	6.5	4.9
Hepatitis C	17.0	18.0	16.6	19.1	23.2	22.7	31.6	32.7	33.0	32.0	31.6	30.7
Others	16.1	17.1	15.9	16.9	15.4	17.1	16.4	16.6	15.2	16.0	13.4	14.0
**Cold ischemia time (%)**												
<8h	40.1	38.7	39.5	43.4	48.6	47.6	41.2	41.5	39.5	40.2	49.0	47.3
8-<12h	35.3	35.5	38.7	35.4	36.9	36.5	35.6	34.9	38.7	38.3	37.3	39.1
>=12h	24.6	25.8	21.8	21.2	14.4	15.9	23.3	23.6	21.7	21.5	13.7	13.6
Missing	26.4	26.7	23.1	24.0	23.6	24.1	24.5	24.9	22.1	24.6	24.2	24.1
**Transplant year (%)**												
1988-1994	24.2	26.7	10.7	13.6	1.9	2.4	17.4	18.2	8.4	7.2	1.0	1.1
1995-1999	25.2	27.6	23.6	22.9	11.3	12.2	25.1	26.2	19.0	19.1	8.4	8.6
2000-2004	24.7	21.8	26.3	26.6	21.6	23.4	25.2	25.0	26.6	27.7	22.2	22.9

2005-2009	16.0	15.0	21.8	19.2	28.8	28.0	18.9	18.4	25.8	24.6	33.0	32.5
2010-2014	7.7	6.9	13.6	13.8	28.2	26.2	10.6	9.5	16.0	16.1	26.9	26.1
2015-2019	2.1	1.9	3.9	3.9	8.2	7.8	2.9	2.7	4.1	5.2	8.5	8.7
**Graft size (%)**												
Whole	92.1	89.1	97.2	95.5	99.6	99.6	94.9	93.8	97.7	98.5	99.6	99.5
Reduced	7.9	10.9	2.8	4.5	0.4	0.4	5.1	6.2	2.3	1.5	0.4	0.5
**Donor CMV (%)**												
Positive	51.8	44.7	61.9	52.9	71.1	59.9	53.3	45.6	62.5	54.8	68.4	61.1
Negative	48.2	55.3	38.1	47.1	28.9	40.1	46.7	54.4	37.5	45.2	31.6	38.9
Missing	14.5	16.0	15.8	17.7	33.8	33.1	21.3	21.7	25.9	26.4	48.9	48.0
**Donor Hypertension (%)**												
No	97.1	98.6	88.7	89.5	82.8	80.5	95.3	97.6	86.7	88.1	83.7	83.2
Yes	2.9	1.4	11.3	10.5	17.2	19.5	4.7	2.4	13.3	11.9	16.3	16.8
Missing	10.2	11.1	6.0	7.1	3.8	3.8	9.6	10.3	6.4	5.3	2.8	3.1
**Donor cause of death (%)**												
Cerebrovascular accident	52.8	28.4	83.2	68.6	82.4	71.9	53.1	29.8	80.3	66.4	83.4	73.2
Other	47.2	71.6	16.8	31.4	17.6	28.1	46.9	70.2	19.7	33.6	16.6	26.8
Missing	10.6	11.2	6.6	7.5	5.0	5.5	9.9	10.6	7.0	5.9	4.0	4.3
**Donation after circulatory death (%)**												
No	99.7	99.5	99.4	99.2	99.7	99.1	99.5	99.1	99.1	98.9	99.5	98.9
Yes	0.3	0.5	0.6	0.8	0.3	0.9	0.5	0.9	0.9	1.1	0.5	1.1
Missing	9.1	10.2	4.9	5.9	1.8	2.5	8.3	9.0	4.8	3.6	1.6	1.7

Because the unit of analysis was person-time, rather than person, the characteristics presented are weighted by a factor derived from the number of person-years of observation and number of events. For example, 3.4% of the person-years contributed by female recipients of female donors between 13 and 44 years were from people with congenital liver disease/ biliary atresia as the primary disease.

CMV: cytomegalovirus

Table S5: Median [IQR] MELD scores at transplant by donor-recipient sex combination in CTS and SRTR

	Female recipient		Male recipient	
	**Female donor**	**Male donor**	**Female donor**	**Male donor**
**CTS***	26 [17–33]	28 [17–34]	23 [15–31]	22 [15–29]
**SRTR****	21 [14 - 31]	23[15-33]	19 [12- 29]	19 [13-29]

*MELD in CTS (median with IQR)—restricted to 2006 or later; missing in 66% of patients

**MELD distributions for the SRTR cohort restricted to those transplanted in 2003 or later.

Table S6: Sensitivity Analysis Examining the association between Donor Sex and Mortality in the SRTR cohort in Multivariable Models Including and Excluding Donor: Recipient Weight Ratio

	**With Donor:Recipient Weight Ratio HR (95% CI)**	**Without Donor:Recipient Weight Ratio HR (95% CI)**
**First 3 Months**		
Female Recipient		
Donor Age		
13-44	0.98 (0.88-1.10)	0.99 (0.89-1.10)
45-59	0.91 (0.78-1.06)	0.91 (0.78-1.06)
60+	0.97 (0.79-1.19)	0.97 (0.79-1.19)
Male Recipient		
Donor Age		
13-44	1.10 (1.01-1.21)	1.10 (1.01-1.21)
45-59	1.05 (0.93-1.18)	1.0.5 (0.93-1.18)
60+	1.20 (1.02-1.40)	1.20 (1.03-1.40)
**After 3 Months**		
Female Recipient		
Donor Age		
13-44	0.96 (0.92-1.01)	0.96 (0.92-1.01)
45-59	0.89 (0.83-0.96)	0.89 (0.84-0.96)
60+	0.96 (0.88-1.04)	0.96 (0.88-1.05)
Male Recipient		
Donor Age		
13-44	1.05 (1.01-1.09)	1.05 (1.02-1.09)
45-59	0.97 (0.92 -1.01)	0.97 (0.93-1.01)
60+	0.97 (0.92-1.03)	0.98 (0.92-1.04)

Hazard ratios show the risk in female donors compared with male donors in SRTR. Models were adjusted for age at transplant (piecewise linear with 3 pieces: 13-<20, 20-<35, 35+y), recipient race (HepC as reference), primary disease, cold ischemia time (continuous linear), transplant year (continuous linear), whole liver vs other, donor HTN, donor CMV, donor cerebrovascular accident as well as donor:recipient weight ratio.

Figure S1a: Unadjusted association between donor sex and mortality in the first 3 months post-transplant for female and male recipients.

<u>Female recipients</u>

Donor age 13-44

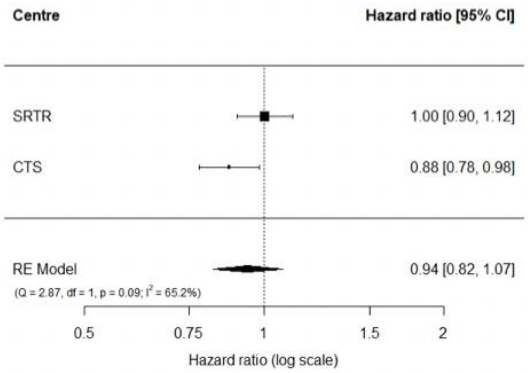

Donor age 45-59

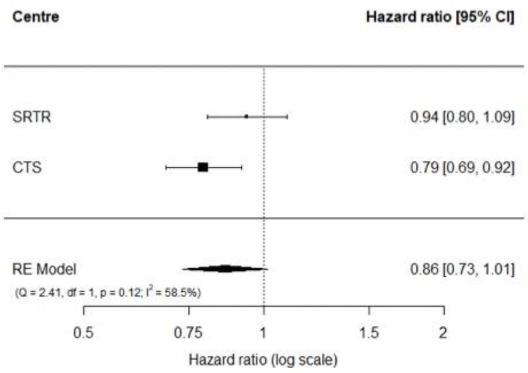

Donor age 60+

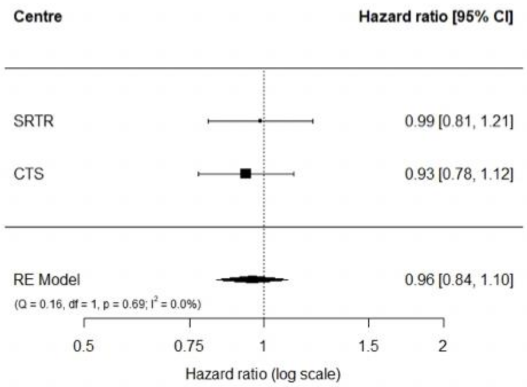

<u>Male recipients</u>

Donor age 13-44

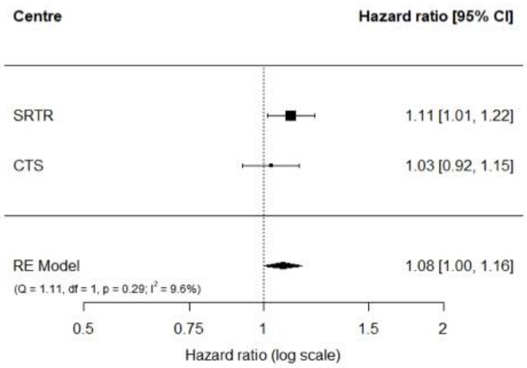

Donor age 45-59

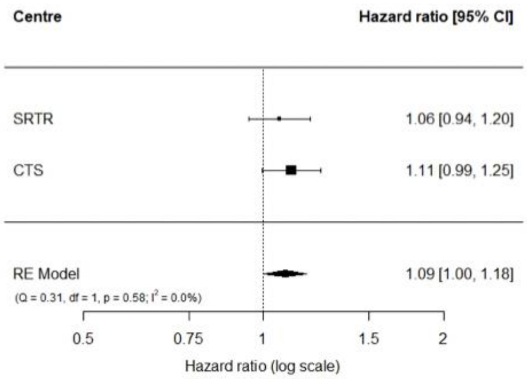

Donor age 60+

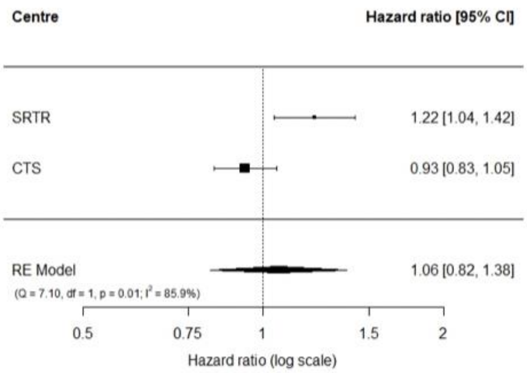

Figure S1b: Unadjusted association between donor sex and mortality in the time subsequent to 3 months post-transplant for female and male recipients.

<u>Female recipients</u>

Donor age 13-44

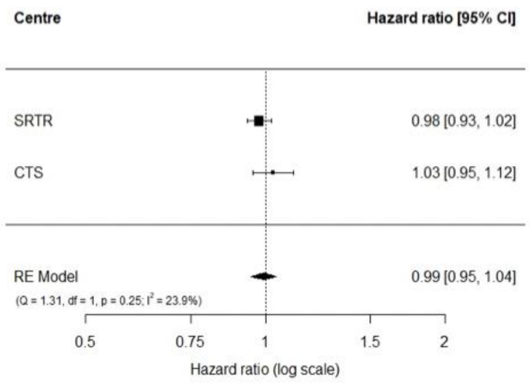

Donor age 45-59

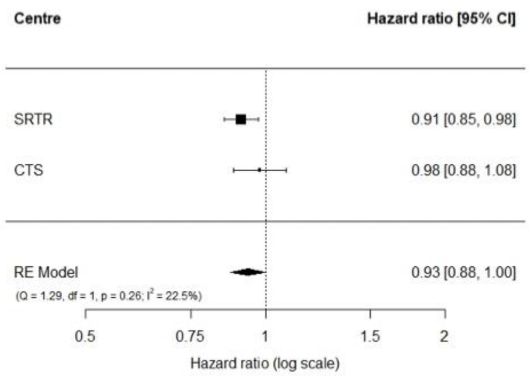

Donor age 60+

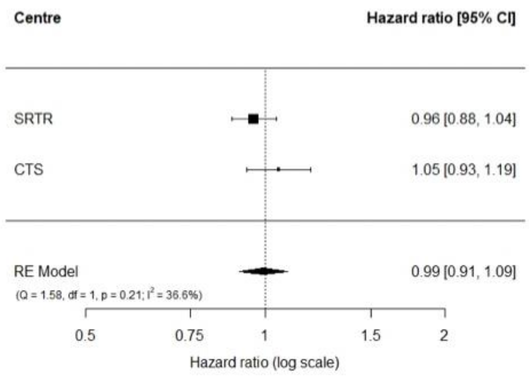

<u>Male recipients</u>

Donor age 13-44

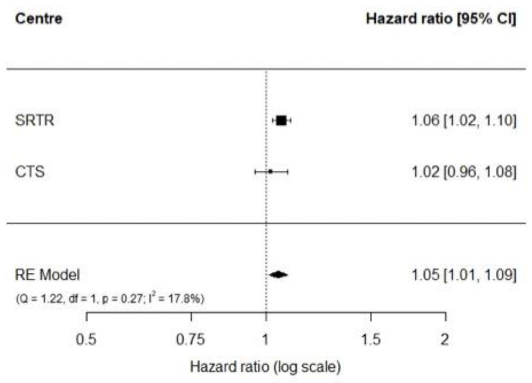

Donor age 45-59

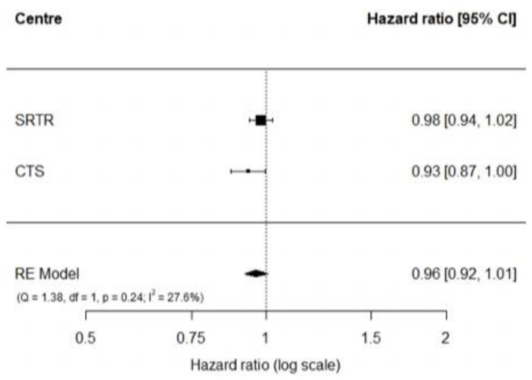

Donor age 60+

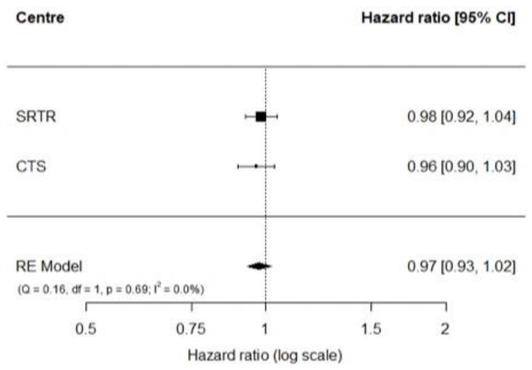

Figure S2: Relative hazards of mortality and of graft failure in female and male recipients of female versus male liver donors

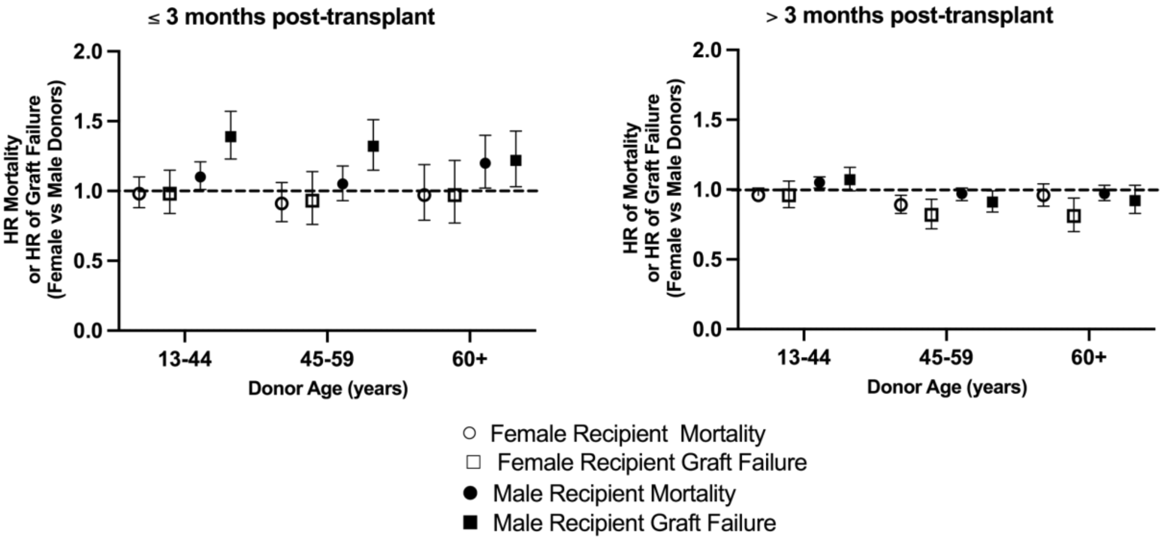

FIGURE LEGENDS

Figure S1a: Unadjusted association between donor sex and mortality in the first 3 months post-transplant for female and male recipients. Forest plots show the unadjusted hazard ratios (HR) for mortality in female and male recipients of female relative to male donors of each donor age. HR are shown separately for the SRTR and CTS cohorts, as well as the pooled HR from the meta-analysis.

Figure S1b: Unadjusted association between donor sex and mortality in the time subsequent to 3 months post-transplant for female and male recipients. Forest plots show the unadjusted hazard ratios (HR) for mortality in female and male recipients of female relative to male donors of each donor age. HR are shown separately for the SRTR and CTS cohorts, as well as the pooled HR from the meta-analysis.

Figure S2: Relative hazards of mortality and of graft failure in female and male recipients of female versus male liver donors. Plots show the relative hazards of mortality and the relative hazards of graft failure in female and male recipients of female versus male liver donors in the first 3 months post-transplant (left) and in time subsequent to the first 3 months (right). Female recipients are shown separately from male recipients.
